# Protocols and Features of Goal-Setting-Based Intervention for Frail Older Adults in Community Exercise Facilities

**DOI:** 10.3390/ijerph20021615

**Published:** 2023-01-16

**Authors:** Masashi Yamashita, Yuki Mashizume, Kento Yama, Shun Sasaki, Daiki Uehara, Kentaro Kamiya

**Affiliations:** 1 Division of Research, ARCE Inc., Sagamihara 252-0306, Japan; 2 Department of Rehabilitation Sciences, Graduate School of Medical Sciences, Kitasato University, Sagamihara 252-0329, Japan; 3 Department of Rehabilitation, Kitasato University Hospital, Sagamihara 252-0329, Japan; 4 Department of Rehabilitation, Kitasato University School of Allied Health Sciences, Sagamihara 252-0373, Japan; 5 Division of Health Promotion, ARCE Inc., Sagamihara 252-0306, Japan

**Keywords:** rehabilitation, goal-setting, frailty domain, protocol

## Abstract

Appropriate goal setting for frail older adults is important because it drives effective rehabilitation. However, more insights into the types and degrees of frailty and goal-setting trends should be obtained. We conducted a multicenter prospective study to qualitatively examine the relation between each frailty domain (physical, social, and cognitive) and the goals of 201 subjects (median age: 79, 43.8% male) who began rehabilitation at a long-term care prevention facility. Goal setting was determined by the specific, measurable, achievable, relevant, and time to goal (SMART) framework up to three months, categorized according to the International Classification of Functioning, Disability, and Health. The results showed that approximately 90% of the subjects had frailty in at least one domain, and about half had frailties in two or more domains. In total, 176 (87.6%) subjects had goals corresponding to activities and participation. The tendency to set goals to improve mobility was confirmed when the number of overlapping frailties was high, especially those in the physical and social domains. Those with milder frailties were more likely to establish goals targeting improvements in community, social, and civic life. These findings will lead to the development of practical goal-setting guidelines for frail older adults.

## 1. Introduction

Japan has the world’s oldest population, and its burden of caregiving continues to increase. Although the long-term care insurance system was introduced in 2000 to aid in the maintenance of the physical functioning and activity levels of frail older people and to reduce the burden of caregiving [1], this approach to disability prevention remains incomplete and requires further revision, especially in the area of correcting frailty [2]. Frailty is caused by the aging process and the accumulation of age-related deficits across physical, social, and psychological health domains. Without the appropriate interventions, it will contribute to an increased risk of nursing care [3] and a higher risk of death [4]. Many long-term care interventions for frail older adults have been reported [5]. Although they have shown efficacy in maintaining physical function and activity, they have not sufficiently improved or mitigated adverse events [6].

In particular, rehabilitation for frail older adults is a long-term process, so it may be difficult for them to maintain their motivation. In response to these problems, we focused on rehabilitation based on goal-setting. Goal-setting-based rehabilitation effectively improves the quality of life and self-efficacy, although more evidence of this finding is required [7]. Therefore, we considered it appropriate to motivate frail older adults to continue exercising through appropriate goal setting. However, the trends in rehabilitation goals set by older adults with frailties and the associations between frailty and goal setting have not been sufficiently verified. Moreover, what methodology should be used to evaluate the goal-based rehabilitation process, how and what goals are set by not only the patients but also the frail older adults, and how this knowledge can be translated into specific workflows are questions that remain unclear (in this paper, “patient” means people who participated in previous studies conducted in hospitals and clinics) [8].

If associations between frailty and goal setting could be shown according to a clear protocol, it may be possible to create rehabilitation interventions for frail older adults, based on their wishes and goals, and to mitigate frailty or avoid future adverse events. Therefore, in this paper, we describe the rehabilitation protocol at our long-term care prevention exercise facility and qualitatively examine the association between older subjects’ frailty and initial goal setting (in this paper, “subject” means people who participated in this study or another previous study in the community).

## 2. Materials and Methods

### 2.1. Overall Study Project

This present study was designed as the first report of a multicenter prospective observational study. The entire observational study will initially include up to 500 consecutive subjects enrolled from September 2019 onward. The research was approved by the Ethics Committee of Kitasato University Allied Health Sciences (2020-028) and is being conducted in accordance with the Declaration of Helsinki. Each user of the facility received a comprehensive explanation of the study in the contract document at the time of initial use (in this paper, “user” means people using exercise facilities). An overview of the comprehensive prospective study was also published in a publicly available University Hospital Information Network (UMINCTR, unique identifier: UMIN000043109), and information about the research was made available on the facility’s website and within the facility via an opt-out form, which included information about the option to drop out.

As major physical function indices to grasp users’ physical conditions and estimate the inverse feasibility of the performance recovery [9], gait speed [10], grip strength [11], the Short Physical Performance Battery (SPPB) [12], quadriceps’ isometric strength [13], arm [14] and calf [15] circumferences, and body composition [15] are evaluated throughout the study protocol. The users’ conditions are measured periodically (every 3–6 months) through major physical function tests and several questionnaires, following each institution’s standards.

The entire project’s primary outcome is all-cause death within five years of follow-up after the end of facility use. New diagnoses, hospitalizations, and other adverse events will also be investigated. A telephone survey will be conducted after the end of facility use, and cases that cannot be confirmed for more than one year will be excluded. On the other hand, the primary goal of the in-facility rehabilitation is to improve the user’s quality of life as assessed by the EQ-5D-5L (a secondary outcome due to the study design) [16]. Other outcomes include the degree of goal attainment, as assessed by the change in the Canadian Occupational Performance Measure (COPM) score [17], improvements in major physical function test results, physical activity outside of the facility, and the change in the physical frailty domain.

### 2.2. Study Population

The study’s subjects were users of either of the two exercise facilities for the purpose of preventing the need for nursing care between September 2019 and March 2022 and participated in at least one rehabilitation program. Users who declined to participate in the study, discontinued use without setting a rehabilitation goal, and were unable to undergo comprehensive frailty assessments at all for any reason were excluded.

### 2.3. Rehabilitation Protocol and Setting Goals for Rehabilitation

The exercise facilities in this study provided rehabilitation services every half day on weekdays. Eligibility to use this exercise facility due to some disease or frailty with long-term care insurance coverage was approved by the government. The facility’s acceptance criteria for users were those who were able to walk indoors independently with the use of aids (functional independence measure: levels 5–7) and were independent in toilet behavior and control of elimination (functional independence measure: levels 6–7). Transportation to and from the facility was available for users who lived far away or were frail. Each rehabilitation session lasted for three hours and consisted of the following elements: a 10-min vitality check and warm-up exercises, approximately 2.5 h of training according to each subject’s physical condition (7–10 different exercise programs, each lasting 10–20 min), 10-min vitality check and cool-down exercises, and a short, approximately 15-min lecture on health. A maximum of 10 users were included in each session (20 users per day) under the supervision of 3–4 professionals (more than 90% were physical or occupational therapists). The content of the exercise program differed for each user. Users could use the facility from one to five days a week, depending on their wishes and long-term care insurance grade.

**Figure 1:**
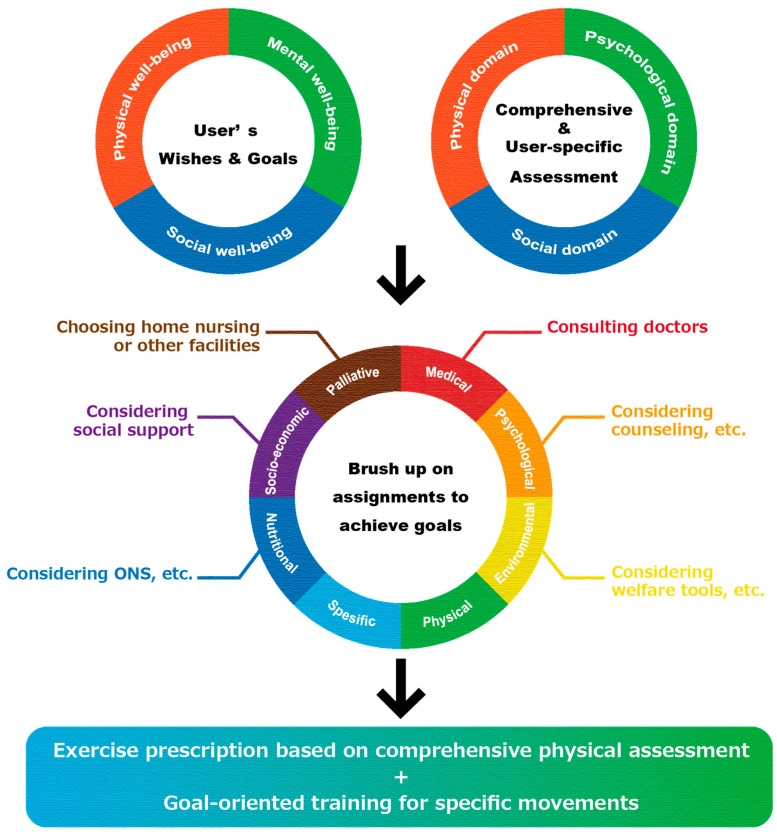
Conceptual illustration of the rehabilitation protocol. First, the user’s wishes and goals were heard. Then, a comprehensive frailty assessment was conducted. After elucidating the issues related to the subject’s wishes and goals, an intervention program was designed to target issues related to physical functions and specific movements. ONS = oral nutritional supplement.

The rehabilitation protocol used in this study is schematically depicted in Figure 1. In the beginning, users’ wishes for rehabilitation were heard, and a comprehensive frailty assessment was conducted. Physical function assessments and anamnesis about the users’ medical, pharmacological, and living conditions were also conducted. The users’ wishes were made by applying the concept of balanced health [18] based on well-being, consisting of physical, social, and psychospiritual components, and were heard according to the specific, measurable, achievable, relevant, and time to goal (SMART) framework [19]. The time to goal was based on a three-month period. A goal-setting decision-aid tool, such as Aid for Decision-making in Occupation Choice (ADOC), was used as needed [20]. Then, based on the users’ wishes and the results of each frailty domain assessment, the issues that needed to be addressed to achieve the goals were clarified. At this time, we contacted specialists in each field to address any issues that were difficult to resolve at the exercise facility.

After the issues were clarified, an exercise list to be implemented at this facility was developed. We kept in mind that this list should consist of a multi-component exercise program, such as resistance training, aerobic exercises, and balance exercises, based on the exercise statement for older adults [21,22]. Furthermore, exercise risk management and discontinuation criteria followed the guidelines of the Japanese Association of Cardiac Rehabilitation [23] and the Japanese Association of Rehabilitation Medicine [24], based on vital checks, visual checks, palpation, and medical interviews, without the use of medical equipment.

Regarding the interventions, we adopted the basic concept of a goal-oriented approach. A top-down approach to goal setting is crucial, especially for older adults with multiple morbidities [25] and several chronic diseases that may cause various health problems. Thus, in our clinical practice, we provide exercise therapy according to each user’s goals and by referring to task-oriented training, which is mainly implemented to rehabilitate patients with paralyzed upper extremities after a stroke [26]. The exercises carried out at our facilities can be directed toward each goal, are repetitive, and are associated with adequate levels of difficulty that constitute functional challenges [27].

The process of setting specific goals and determining specific exercises according to the subject’s wishes was based on the concept of shared decision making (SDM) [28]. Everything, from goal setting to exercise list planning, was carried out based on the agreement between the user and the specialist [29].

### 2.4. Assessment of Each Frailty Domain and International Classification of Functioning, Disability and Health (ICF) Classification

Before planning an exercise program, a comprehensive frailty assessment was performed. The assessment and scoring of each frailty domain were performed based on a previous report by Mastue et al. [30]. Specifically, we assessed the physical, social, and psychological (cognitive) domains. The physical domain was determined according to the revised Japanese version of the Cardiovascular Health Study, based on Fried’s frailty criteria: (1) low muscle strength determined by grip strength, (2) slow to usual gait speed, (3) decline in physical activity, (4) loss of body weight, and (5) feeling fatigued. A score of 3 or higher was considered physical frailty [31]. The social domain was assessed by Makizako using five items: a decrease in (1) going out, (2) daily conversations, (3) visiting a friend’s house, (4) being useful to anyone else, and (5) living alone. A score of 2 or higher was considered social frailty [32]. The psychological (cognitive) domain was determined using the Mini-Cog test, which consists of a three-word post-recall and a two-point clock drawing test [33]. Cognitive dysfunction was determined if the total score was less than 2 points. Details of each frailty domain are provided in Table S1.

The content of the initial goals was converted into a format that could be analyzed by linking the goal concepts to the ICF [34,35]. Two authors (Y.M. and K.Y.) independently classified all described goals by linking them to specific ICF codes in accordance with the ICF linking rules [36]. The ICF codes consist of major items at the first level and minor items at the second level. When there were cases in which the classification was unclear or the target content overlapped, we checked these with another author (M.Y.) and re-categorized them.

### 2.5. Statistical Analysis

Continuous and nominal variables are shown as medians (interquartile range) and *n* (%), respectively. All analyses were performed on complete data, and missing rates are presented in the descriptive statistics. The number of overlapping frailty domains was determined for subjects for whom all frailty domains could be investigated. Then, an area-proportional diagram was used to visualize the number of subjects in the discontinuous and overlapping regions of each frailty domain using the eulerr package version 6.0.0 of R (https://cran.r-project.org/package=eulerr) (accessed on 17 September 2022) with complete data. R version 4.0.2 was used. Additionally, the frequency distribution was used to visually examine how the number of overlaps in each frailty domain changed with age (stratified to <65 years, <75 years, <85 years, and ≥85 years) using JMP (ver. 16.1, SAS Institute Inc., Cary, NC, USA).

In this study, a qualitative approach was chosen to analyze the initial goals. For the analysis about the relation between goal setting and each frailty domain, a bubble chart was generated using KH Coder (ver. 3, Ritsumeikan University, Kyoto, Japan) to visually verify the frequencies and associations. The first level of ICF codes was used to generate the bubble chart. In this software, the strength of the correlations between the goal-setting categories and each frailty domain is indicated by the Pearson correlation coefficient, with positive correlations shown in red and negative correlations in blue. The color intensity indicates the degree of the correlation coefficient, and the percentages of the cross-tabulation are visually indicated by the sizes of the bubbles.

## 3. Results

### 3.1. Subjects’ Characteristics

In total, 201 subjects were enrolled in the study. The subjects’ backgrounds are shown in Table 1. Their median age was 79 (interquartile range: 74–82), and 43.8% of the subjects were male. Those who used the facility once a week, twice a week, and three or more times respectively accounted for 77.1%, 21.4%, and 1.5% of all subjects. Among all subjects, 40.6% suffered physical frailty (*n* = 71/175), 24.7% suffered cognitive dysfunction (*n* = 41/166), and 75.8% suffered social frailty (*n* = 122/161).

### 3.2. Prevalence of Each Frailty Domain and Overlapping Frailties

**Figure 2:**
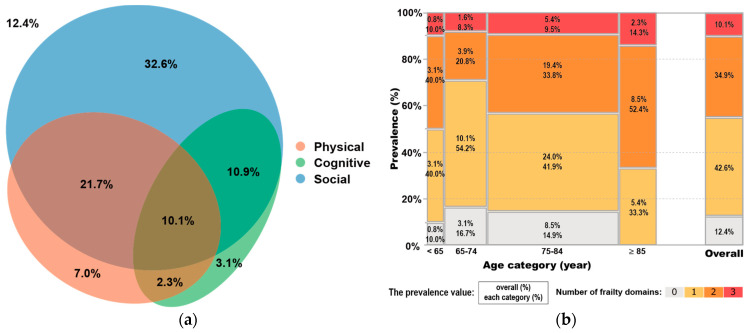
(**a**) The prevalence of each frailty domain according to the physical domain (orange), the social domain (blue), and the cognitive domain (green), as well as their overlaps according to the complete data. (**b**) The prevalence of overlapping frailties is stratified by age according to the overall category (above) and each age category (below).

The proportions of each frailty domain are shown in Figure 2a (using complete data). Only 12.4% of all subjects had no frailty at all. Moreover, each frailty domain showed significant overlap, and approximately 50% of the subjects suffered in two or more frailty domains. Figure 2b shows the percentage of overlapping frailties by age. More than 70% of the subjects were over 75 years old, a significantly older population, and 7.8% of the subjects were younger than 65. Approximately 90% of the subjects across all age groups were found to have some type of frailty domain. The number of overlapping frailties increased with age, and two-thirds of the subjects aged 85 years and older had overlapping frailties in two or more domains. All subjects who were over 85 years old had frailties in one or more domains.

### 3.3. Setting Goals According to the Subjects’ Wishes

A summary of the subjects’ goal setting is presented in Table S2. The subjects’ goals that were found to correspond to the second level of the ICF category were divided into 31 types. A total of 176 (87.6%) subjects had goals in the ICF category corresponding to “d: Activities and Participation”. Another 10% of the subjects had “b: Body Functions” as their goal, but none had other factors (“s: Body Structures” and “e: Environmental Factors”). The most frequent goal in general was “d460: moving around in different locations”. About 40% of all subjects (*n* = 80) had a target of “d4: moving”.

### 3.4. Relation between Goal Setting and Frailty Domain

**Figure 3:**
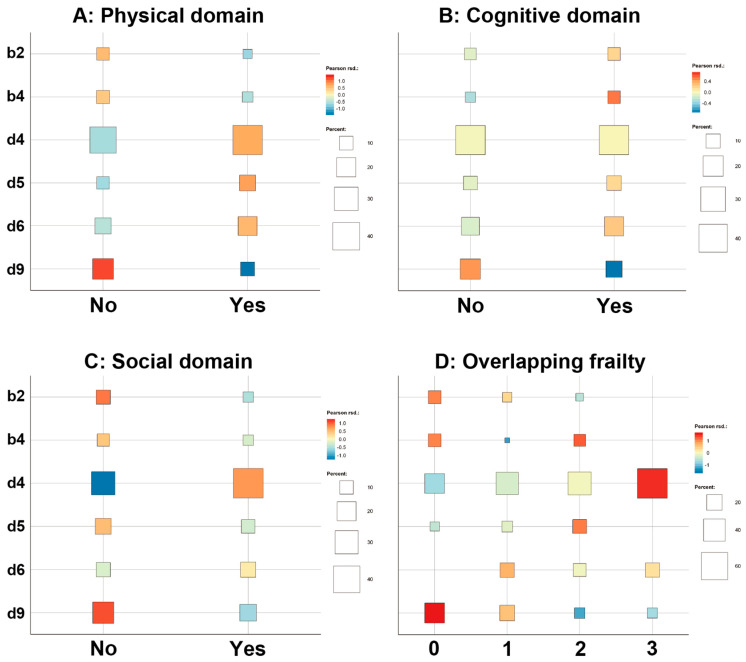
Relationship between goal setting and each frailty domain ((**A**): physical, (**B**): cognitive, (**C**): social), and overlapping frailty domains (**D**).

The main observational goal of this study, the association between each frailty domain and goal setting, is shown in Figure 3. While a high percentage of the subjects with physical frailty preferred to work toward goals that fell under “d4: mobility”, a small number of the subjects without physical frailty also set goals that fell under “d9: community, social, and civic life”. No clear difference in goal setting was observed between subjects with and without cognitive dysfunction. However, the same trend was observed in subjects with and without social frailty as in those with physical frailty. When the number of overlapping frailties was small, the subjects tended to target “d9: community, social, and civic life”, and when the number of overlapping frailties was large, they tended to target “d4: mobility” or “d5: self-care”. Most subjects with frailties in all domains targeted “d4: mobility”.

## 4. Discussion

To our knowledge, this study was the first to capture the subjects’ wishes and goal trends according to each frailty domain in the long-term care prevention facility setting, and several key findings were observed. First, almost 90% of facility users already had frailties in at least one domain, and about half had two or more overlapping frailties. Second, most subjects wanted to set goals related to activities and participation, especially in relation to mobility, and none of them set goals related to body structure or environmental factors, primarily mental or psychological factors. Third, the subjects’ tendency to target activity and participation when they suffered from physical frailty, social frailty, or a high number of overlapping frailties was confirmed. In contrast, the number of subjects who set community, social, and civic life goals increased when the degree of frailty was mild. Currently, few exercise facilities provide rehabilitation services based on precise goal setting for frailty, which is considered one of the major strong points of this study. These findings will lead to the development of practical goal-setting guidelines for frail older adults, thus contributing to the improvement of motivation for and efficacy of rehabilitation implementation.

A previous study that most closely resembles our validation study examined meaningful activities (activities that individuals consider essential in their daily lives), which are similar to their wishes or hopes, according to each frailty domain and desires using the ADOC [37]. This previous study’s results showed that the physical domain of frailty affected choices in physical activity, the cognitive domain influenced choices in cognitive activity, and multiple domains had impacts on choices regarding social activities. The most significant difference between this previous report and ours is whether the subjects’ wishes were targeted as the goal of rehabilitation. At first glance, the multiple domain-related results of the previous study are at odds with our results, but they can be interpreted as follows. The ultimate goal of a patient with overlapping frailties is to participate in society. However, as part of this process, the achievable goal of rehabilitation is for the patient to first become active in different locations. In this sense, this study suggests the importance of setting rehabilitation goals according to the users’ wishes while following more specific guidelines in making decisions.

A meta-analysis of frailty among community-dwelling older adults in Japan reported a frailty prevalence of 7.4% [38]. A subsequent nationwide survey in Japan also showed a prevalence of 8.7% for physical frailty [39]. In contrast, in the facilities surveyed, almost all subjects in the present study had frailties in one or more domains, and approximately 40% suffered from physical frailty. These ratios are rather similar to those found in Japan’s large nationwide study of patients requiring inpatient care, such as heart failure patients [30]. This previous study showed that overlapping frailty domains resulted in a higher risk of all-cause death. Another previous study on community-dwelling older adults also indicated that the overlapping of several frailty domains promoted adverse events, such as institutionalization and mortality [4]. Based on these results, the current study accurately captured the frail population among community dwellers. In the future, it will be necessary to establish effective interventions for this high-risk, frail population.

Yoshimi et al. conducted a cluster nonrandomized controlled trial of a goal-setting-based intervention for subjects with frailties [40]. The subjects of this previous study showed some improvement in their quality of life and frailty, as determined by a self-administered questionnaire. However, a large randomized controlled trial reported that a multicomponent intervention maintained mobility and physical function but did not reduce any adverse events among older adults with physical frailty, as determined by SPPB [6]. Based on these previous studies, the effectiveness, indications, and limitations of the goal-setting-based multicomponent intervention, which will be validated through this project, in improving frailty need careful consideration. In addition to maintaining and improving measurements in the gym and reducing adverse events, there is a need to assess frailty using wearable accelerometers or smartphone applications, with a focus on improving daily living in the future.

Most subjects in this study were actively requested to use the facility, and their personalities, living environments, health literacy levels, and lifestyles may support the results of this study [41]. Recent studies showed that limited health literacy was associated with an increased risk of future frailty [42,43]. However, we observed that many older adults who used the exercise facilities and participated in this study appeared to have high health literacy but had frailties due to aging or some other disease. Of course, although it is important to prevent the future onset of frailty in older adults with low health literacy, we believe that it would also be essential to support older adults with frailty who wish to lead healthier lives than they have now. As a perspective, our entire project that aims to provide rehabilitation based on individual wishes and goals by regularly assessing the subjects’ goals, their degree of frailty, and their physical functions at a community exercise facility for frail older adults will provide valuable information to support them in achieving their aspirations to lead healthier lives.

Although this study offers several significant findings, some limitations exist. The purpose of this report is to specify the protocol and subject demographics, but a sufficient sample size has not yet been reached. Although the analysis should have been performed up to the second level of ICF codes, there was a risk that the number of subjects per variable would become infinitely small. Furthermore, although this project is a multicenter prospective study in the versatile setting of a community exercise facility, it focuses only on a few long-term care and disability prevention facilities in Japan. Nevertheless, this study would provide valuable findings about a frail population motivated to exercise, so it has sufficient novelty. To demonstrate the generalizability of this study’s results, additional validation at other regional facilities should be desired. Finally, in the current report, we only perform a qualitative analysis and do not specify some quantitative variables, such as COPM, for which measurements are ongoing as the project is continuing. For example, COPM is a simple and easy-to-interpret indicator with high test-retest reliability, so it will offer relevant information for goal-based rehabilitation and provide feedback on the achievement of each user’s goals, specifically those of older adults [44]. In the future, it will be necessary to conduct an analysis that adds a value measure, such as COPM, to each goal set.

## 5. Conclusions

More than 90% of the subjects using Japanese exercise-focused long-term care prevention facilities in this study suffered from at least one form of frailty in one domain, and about half had multiple overlapping frailties. Furthermore, 87.6% of the subjects set rehabilitation goals related to activity and participation, while approximately 10% of the subjects set goals related to body function recovery. It was also confirmed that they tended to aim for goals related to community, social, and civic life when the number of overlapping frailty domains was low and to target mobility or self-care when the number of overlapping frailty domains was high. Further verification is needed to determine whether rehabilitation based on such goals will contribute to the mitigation of frailty, improvements in physical function and quality of life, or the suppression of adverse events.

## Figures and Tables

**Table 1:** Study subjects’ characteristics.

(*n* = 201)	All Subjects	Missing Data
Age, year	79 (74–82)	-
Male, *n* (%)	88 (43.8)	-
BMI, kg/m^2^	22.6 (22.1–24.6)	5 (2.5)
Frequency of use, *n* (%)		-
- 1/week	155 (77.1)	
- 2/week	43 (21.4)	
- 3 or more/week	3 (1.5)	
Disease, *n* (%)		-
- Orthopedic disease	124 (61.7)	
- Cerebrovascular disease	54 (26.9)	
- Cardiovascular disease	37 (18.4)	
- Cancer	28 (13.9)	
- Intractable disease ^1^	12 (6.0)	
Frailty domain, *n* (%)		
- Physical frailty	71 (40.6)	26 (12.9)
- Cognitive dysfunction	41 (24.7)	35 (17.4)
- Social frailty	122 (75.8)	40 (19.9)

Notes: The results show the median (interquartile range) or *n* (%). BMI = body mass index. ^1^ Intractable diseases included Parkinson’s disease, spinocerebellar degeneration, and multiple system atrophy.

## Data Availability

The data from this study are not publicly available for privacy reasons but can be requested from the corresponding author if necessary.

## Supplementary Materials

The following supporting information can be downloaded at https://www.mdpi.com/article/10.3390/ijerph20021615/s1: Table S1: The definition of each frailty domain. Table S2: The number of applicable persons for each ICF category.
