# Supplementary material for: Protocols and Features of Goal-Setting-Based Intervention for Frail Older Adults in Community Exercise Facilities
Source: Int J Environ Res Public Health. 2023 Jan 16;20(2):1615. doi: 10.3390/ijerph20021615 (PMC9865582; doi:10.3390/ijerph20021615)
Supplement: Supplementary file 1 [file ijerph-20-01615-s001.zip › ijerph-2104955-supplementary.pdf]

Supplementally Table S1: The definition of each frailty domain

**Physical domain (robust: 0 points, most frail: 5 points, 3 or more is defended as the physical frailty.)**	
**[Sub-items]**	**[allotment criteria]**
low muscle strength	< 28 kg for men, < 18 kg for women
slow usual gait speed	< 1.0 m/s
decline in physical activity	1) Do you engage in moderate levels of physical exercise or sports aimed at health?
	2) Do you engage in low level of physical exercise aimed at health?
loss of body weight	Have you unintentionally lost 2 or more kg in the past 6 months?
feeling fatigued	In the past 2 weeks, have you felt tired without a reason?
**Social domain (robust: 0 points, most frail: 5 points, 2 or more is defended as the social frailty.)**	
**[Sub-items]**	**[allotment criteria]**
Yes = 1, No = 0	A decrease in going out compared with last year
Yes = 0, No = 1	Daily conversations with someone
Yes = 0, No = 1	Visiting a friend's house sometimes
Yes = 0, No = 1	Being useful to anyone else (friends or family)
Yes = 1, No = 0	Living alone
**Cognitive domain (robust: 5 points, most frail: 0 points, 1 or less is defended as the cognitive decline.)**	
**[Sub-items]**	**[allotment criteria]**
Three-word registration	-
Clock drawing test	Correct answer = 2 points, Incorrect answer = 0 points
Three-word recall	1 point for each word (max: 3 points)

Supplementary Table S2: The number of applicable persons for each ICF category

ICF classification					*n*	%
b	b1	specific mental functions	b152	emotional functions	1	0.5%
	b2	hearing and vestibular functions	b240	sensations associated with hearing and vestibular function	9	4.5%
		pain	b280	sensation of pain	5	2.5%
	b4	additional functions and sensations of the cardiovascular and respiratory systems	b455	exercise tolerance functions	12	6.0%
			b465	additional functions and sensations of the cardiovascular and respiratory systems, other specified and unspecified	1	0.5%
	b7	functions of the joints and bones	b710	mobility of joint functions	1	0.5%
		muscle functions	b730	muscle power functions	1	0.5%
		movement functions	b770	gait pattern functions	1	0.5%
d	d4	changing and maintaining body position	d410	changing basic body position	1	0.5%
		carrying, moving and handling objects	d430	lifting and carrying objects	1	0.5%
		walking and moving	d450	walking	11	5.5%
			d455	moving around	3	1.5%
			d460	moving around in different locations	39	19.4%
			d465	moving around using equipment	20	10.0%
		moving around using transportation	d470	using transportation	5	2.5%
	d5	self-care	d530	toileting	1	0.5%
			d540	dressing	1	0.5%
			d550	eating	1	0.5%
			d570	looking after one's health	14	7.0%
			d599	self-care, unspecified	2	1.0%

	acquisition of necessities	d620	acquisition of goods and services	20	10.0%
		d630	preparing meals	1	0.5%
d6	household tasks	d640	doing housework	8	4.0%
		d649	household tasks, other specified and unspecified	1	0.5%
	caring for household objects and assisting others	d650	caring for household objects	3	1.5%
d7	particular interpersonal relationships	d750	informal social relationships	1	0.5%
		d770	intimate relationships	1	0.5%
d8	work and employment	d845	acquiring, keeping and terminating a job	2	1.0%
		d910	community life	7	3.5%
d9	community, social and civic life	d920	recreation and leisure	26	12.9%
		d930	religion and spirituality	1	0.5%

b: Body functions (b1: mental functions, b2: sensory functions and pain, b4: functions of the cardiovascular, haemato-logical, immunological and respiratory systems, and b7: neuromusculoskeletal and movement-related functions)

d: Activities and Participation (d4: mobility, d5: self-care, d6: domestic life, d7: interpersonal interactions and relationships, d8: major life areas, and d9: community, social and civic life)
